# Promoting global health transdisciplinary research for planetary health: Towards achieving the 2030 Agenda for Sustainable Development

**DOI:** 10.7189/jogh.13.03007

**Published:** 2023-02-10

**Authors:** Long Tam Pham, Pankaj Kumar, Wirawan Dony Dahana, Duc Hong Nguyen

**Affiliations:** 1 Graduate School of Economics, Osaka University, Osaka, Japan; 2 Adaptation and Water, Institute for Global Environmental Strategies, Kanagawa, Japan; 3 Department of Water Engineering, College of Environment and Natural Resources, Can Tho University, Can Tho, Vietnam

**Figure:**
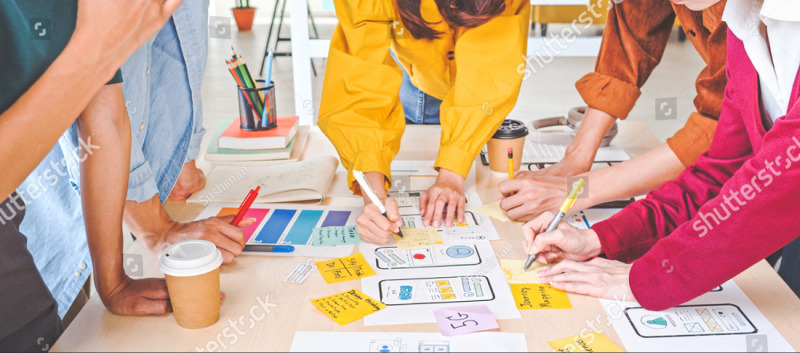
Photo: Young Asian business creative team work together, mobile application software design project. Brainstorm meeting, internet technology, smartphone web app designer, office coworker teamwork concept. Source: Free to use under Shutterstock license (available at https://www.shutterstock.com/image-photo/young-asian-business-creative-team-work-1670616517).

Transdisciplinary research is a type of research that involves the collaboration and integration of multiple disciplines and sectors to address complex and multifaceted problems [1]. In the context of global health, transdisciplinary research is taking control since most of the planet's challenges, such as climate change, environmental degradation, and pandemics, are profoundly interconnected; and thus, a holistic approach is needed [2].

To address global health related issues, a few holistic and multidisciplinary approaches are available among which One Health, EcoHealth, and Planetary Health are the most popular ideas at present [3]. As they all support the basic presumption that humans and other living animals share the same planet and face the same environmental issues, all techniques and methodological approaches to achieve final goal may seem interchangeable. However, the newly coined term “Planetary Health” has revolutionized the subject orientation.

At the beginning, defined as “the collaborative effort of multiple health science professions, together with their related disciplines and institutions” at various scales, One Health prioritizes “the optimal health for people, domestic animals, wildlife, plants, and our environment” [3]. This concept plays as a ground-breaking step to consider not only human beings but also other living species as “One”, that equally needs to be protected. In March 2009, a technical meeting “One World, One Health™: From Ideas to Action” was organized by the Public Health Agency of Canada, to further discuss the key frameworks and recommendations to advance the concepts of One Health, particularly reducing risks of infectious diseases at the Animal-Human-Ecosystems interface. On the other hand, EcoHealth encompasses the well-being of people, animals, and ecosystems, socioeconomic stability, and environmental sustainability. Waltner-Toews suggested that EcoHealth's ultimate goals are “sustainable human and animal health and well-being through healthier ecosystems” [4]. Commitment to nurture the health of humans, animals, and ecosystems and to explore the inextricable linkages between the health of all species and their environments is clearly stated in EcoHealth journal. In that way, Planetary Health is distinctively seen as shifting the focus on human being rather than all species sharing the same rights [5]. This approach is anthropocentric and focuses primarily on human health. Planetary Health is defined as the premium level of health, well-being and equity standards attained through political, economic and social systems development in human society that shapes “the future of humanity and the Earth's natural systems that define the safe environmental limits within which humanity can flourish” [6].

In comparison, One Health, which originated from the field of veterinary medicine, recognizes the interdependence between human, animal, and environmental health. It emphasizes the need for collaboration between different sectors, such as human medicine, veterinary medicine, and environmental science, in order to effectively address health issues that impact multiple domains [7]. Planetary Health, on the other hand, takes a more holistic view, recognizing the interconnections between human health and the health of the entire planet. It seeks to understand and address the impacts of environmental and societal factors on human health, and to find ways to sustainably protect and improve the health of both humans and the planet. While both approaches recognize the importance of interdisciplinary / transdisciplinary collaboration and the need to address health issues at multiple scales, One Health tends to focus more on the direct impacts of animal and environmental health on human health, while Planetary Health takes a broader view, as it involves examining the interconnectedness of human health and the health of natural systems, and how changes in one can affect the other. Planetary Health is a relatively new field, but it has gained increasing attention in recent years as the consequences of climate change, pollution, pandemics, and other environmental problems have become more apparent. Planetary Health aims to understand these issues and find ways to protect and preserve the planet, while also improve human health and well-being. This requires a holistic and interdisciplinary approach involving scientists, policymakers, and other stakeholders from various fields, including public health, environmental science, economics, and more.

Although the concept of Planetary Health has been widely accepted, empirical studies to validate this idea are very few since it is considered as a new science. One example of transdisciplinary research in the field of Planetary Health is the study of the impact of climate change on human health [8]. This research involves integrating knowledge from fields such as climatology, public health, economics, and policy to understand the complex mechanisms through which climate change affects human health and to develop strategies for mitigating and adapting to these impacts. This approach also shows scopes that may not fully addressed within the boundary of One Health. One Health approach may consider the direct impacts of climate change on animal and environmental health and how those impacts may subsequently affect human health in an “one way interaction”, which is cause-and-effect perspective [9], where interaction between different sectors (water, agriculture, food, land, climate etc.) may be unnoticed and hence the magnitude, direction and pattern of these impacts on both human and earth system. Planetary Health approach, on the other hand, considers the long-term consequences of all direct and indirect drivers like climate change, land use changes etc., on global health such as food and water security, and the implications for human health, in which the nexus between related these matters were also taken into account. These more complex and indirect connections between climate change and human health may be less within the scope of One Health, which tends to focus more on the direct links between animal, environmental, and human health. Thus, Planetary Health is closer to the field of sustainability, where the needs of future generation are deliberated as a focal point, as consideration of the impact throughout time is essentially prioritized.

Another example of transdisciplinary research in Planetary Health is the study of the connections between environmental degradation, resource depletion, and human health [10]. It is an interdisciplinary research of environmental science, biology, and public health to understand how environmental degradation and resource depletion can impact human health and develop strategies to address these issues.

With few case studies as mentioned before and practical ground knowledge gap at wider scale, the authors emphasize the need for this research work to be scaled up as an urgent matter, and hence propose following strategies.

First, we need to address common ground across a diversity of academic perspectives in seeking practices for transdisciplinary research for Planetary Health. The widespread use of transdisciplinary approaches to study in the field of sustainability science has led to the development of frameworks that deepen our conceptual knowledge [11]. These successes could also be learned and applied through the specific concept of Planetary Health. In order to synthesize the orientation, world-wide guidance is unquestionably consequential, and there is no better guidance than the Sustainable Development Goals (SDGs) of the United Nations. With 17 goals and 169 targets, research scholars can share their advocacy in finding common goals amongst different sectors and aspects, as long as it is ensured that the shared values they create will contribute to the achievement of SDGs, despite their seemingly broad and disparate research directions.

**Figure 1:**
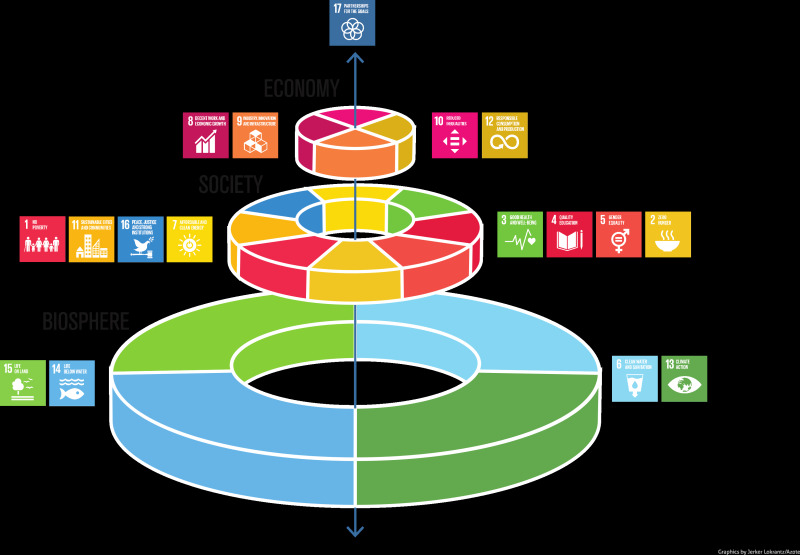
Sustainable Development Goals (SDGs) wedding cake by Stockholm Resilience Center [12].

Second, transdisciplinary research for Planetary Health should be conducted and implemented simultaneously to provide solutions to a variety of global health problems. Taking SDGs as primary guidance is essential but challenging because the goals are separated, and it is necessary to defragment these goals and targets into integrated clusters. It is time for the SDGs wedding cake framework to play a pivotal role in leading experts from different orientations and research fields together and contribute towards the 2030 Agenda for Sustainable Development. **Figure 1** illustrates different SDGs clustered into three large layers: biosphere, society and economy, together with SDG 17 – partnerships for the goals as the core pillar of the spindle. Scholars could consider this framework as an innovative way to connect their research in different aspects, including economic, social, and ecological fields of SDGs.

Third, transdisciplinarity in education, is a key pedagogical methodology for a new science, which is characterized by intrinsic complexity and hybridization of previously separated academic disciplines [13]. Taking a systemic, global, and integrated view may break down traditional disciplinary barriers and create a new organizational structure for different fields. This method incorporates the concept of extensive cross-disciplinary peer review research work, which is especially well-suited to tackle modern difficulties. It is supposed that non-academic stakeholders would join scientists in building knowledge and developing real-world answers to societal issues. The vitality to investigate whether a curriculum geared toward transdisciplinarity in Planetary Health can be successfully implemented at different academic levels and how it may benefit from having students from various backgrounds.

Last but not least, global collaboration and partnership are essential for both human and environmental well-being. Because the current global issues are unlikely to be handled by any sector or in one generation, collective efforts must be founded on cross-sectoral and inter-generational collaboration. More opportunities should be created for early career researchers to join force and deliver innovative ideas. This interprofessional collaboration could be the most effective way to consolidate the partnerships for the goals, which is the ultimate objective of SDG 17.

In conclusion, transdisciplinary research is essential in addressing the complex and multifaceted challenges facing the planet and its inhabitants. For us, the issue is not which of the three holistic approaches – One Health, EcoHealth, or Planetary Health – is more applicable to sustainable development. Instead, the debate is whether the ideas and associated frameworks of One, Eco and Planetary Health should stand as autonomous solutions to the complex nature of global health, as defined by the 2030 agenda for sustainable development, or if it is better to aspire for a growing literature confluence and a broader and stronger commitment to interdisciplinary and transdisciplinary activities around the SDGs. By bringing together experts from multiple disciplines and sectors, transdisciplinary research allows a more comprehensive and holistic approach to understand these challenges, resulting in optimal effective and sustainable solutions. As such, transdisciplinary research will continue to be a vital component of efforts to promote global health and address the many challenges facing our planet.
